# Unvaccinated and Unprepared: A COVID-19 Saga of Diffuse Alveolar Hemorrhage

**DOI:** 10.7759/cureus.77036

**Published:** 2025-01-06

**Authors:** Anam Umar, Muhammad Umer, Hassan Ali, Hamayl Zeeshan, Beena Ahsan

**Affiliations:** 1 Internal Medicine, Ascension St. Vincent's Birmingham, Birmingham, USA; 2 Internal Medicine, Shaikh Khalifa Bin Zayed Al-Nahyan Medical and Dental College, Lahore, PAK; 3 Internal Medicine, Dow University of Health Sciences, Karachi, PAK; 4 Pathology, Henry Ford Health, Detroit, USA

**Keywords:** covid-19, diffuse alveolar hemorrhage (dah), multiplle organ failure, pulmonary critical care, unvaccinated covid-19, veno-venous extracorporeal membrane oxygenation (vv ecmo)

## Abstract

Diffuse alveolar hemorrhage (DAH) is a severe complication that can arise from various conditions, including COVID-19. This case report describes a 37-year-old Caucasian male with mild asthma and obesity who developed diffuse alveolar hemorrhage (DAH) following a COVID-19 infection. Despite initial treatment, his respiratory status deteriorated, leading to intubation and venovenous extracorporeal membrane oxygenation (VV ECMO). Multiple complications followed, culminating in multi-organ failure and his death. This case highlights the complexity of managing DAH in severe COVID-19 and underscores the importance of early recognition and a multidisciplinary approach. Additionally, a literature review compares similar cases to analyze outcomes and management strategies.

## Introduction

Diffuse alveolar hemorrhage (DAH) is a life-threatening emergency characterized by nonspecific signs, symptoms, and abnormal findings on chest imaging, leading to respiratory failure and death [1]. Hemoptysis, anemia, and new lung infiltrates are common presentations, and although hemoptysis is absent in one-third of cases [2], hemorrhagic bronchoscopic bronchoalveolar lavage (BAL) on serial samples confirms the diagnosis [3]. DAH is caused by conditions like vasculitis, thrombocytopenia, post-autologous stem cell transplantation, autoimmune and coagulation disorders, drugs, and infections. Treatment depends on the underlying cause. DAH is also a rare complication of severe COVID-19, particularly in unvaccinated individuals, though the widespread administration of COVID-19 vaccines has reduced the severity of the illness and its complications.

In this report, we present a case of DAH secondary to COVID-19, which led to the unfortunate demise of the patient. This case underscores the critical importance of early recognition and prompt intervention in managing DAH, particularly within the unvaccinated population. By examining the clinical presentation, diagnostic challenges, and therapeutic strategies in this case, we emphasize the need for heightened awareness among healthcare providers. Additionally, this report underscores the broader public health implications of vaccination in preventing severe COVID-19 outcomes.

## Case presentation

A 37-year-old Caucasian male, with a medical history that includes mild exercise-induced allergic asthma, anxiety, obesity, and gastroesophageal reflux disease (GERD), initially presented with worsening shortness of breath and reported being unvaccinated to COVID-19. He had been discharged from the hospital just one day prior after testing positive for COVID-19. Despite receiving treatment with prednisone and azithromycin, his symptoms persisted, leading him to return to the emergency room. He noted that his initial symptoms began a week ago, which he attributed to COVID-19 exposure at work.

**Figure 1:**
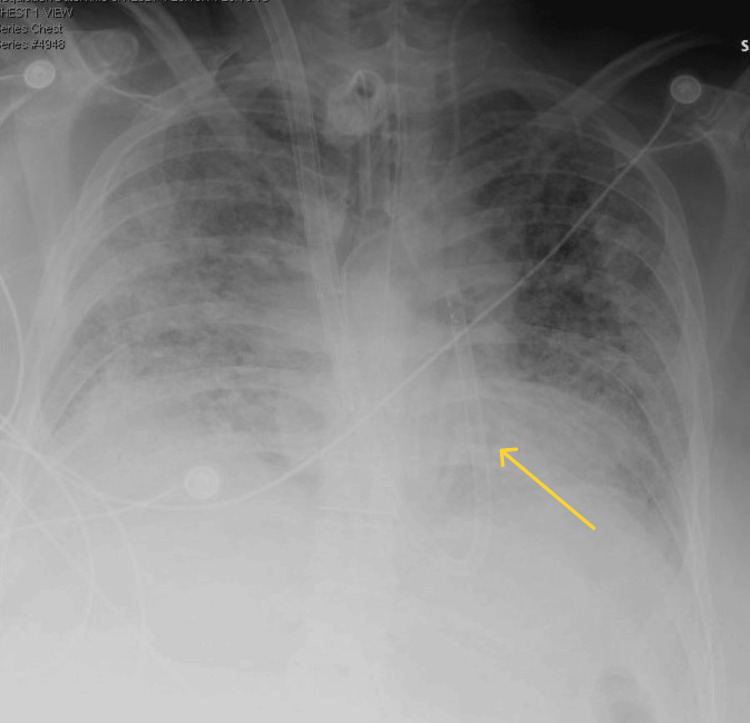
Chest X-Ray The yellow arrow indicates diffuse bilateral parenchymal airspace opacity concerning Acute Respiratory Distress Syndrome (ARDS) versus pulmonary edema. Stable cardiac and mediastinal contours. Small bibasal pleural effusions. No pneumothorax.

**Figure 2:**
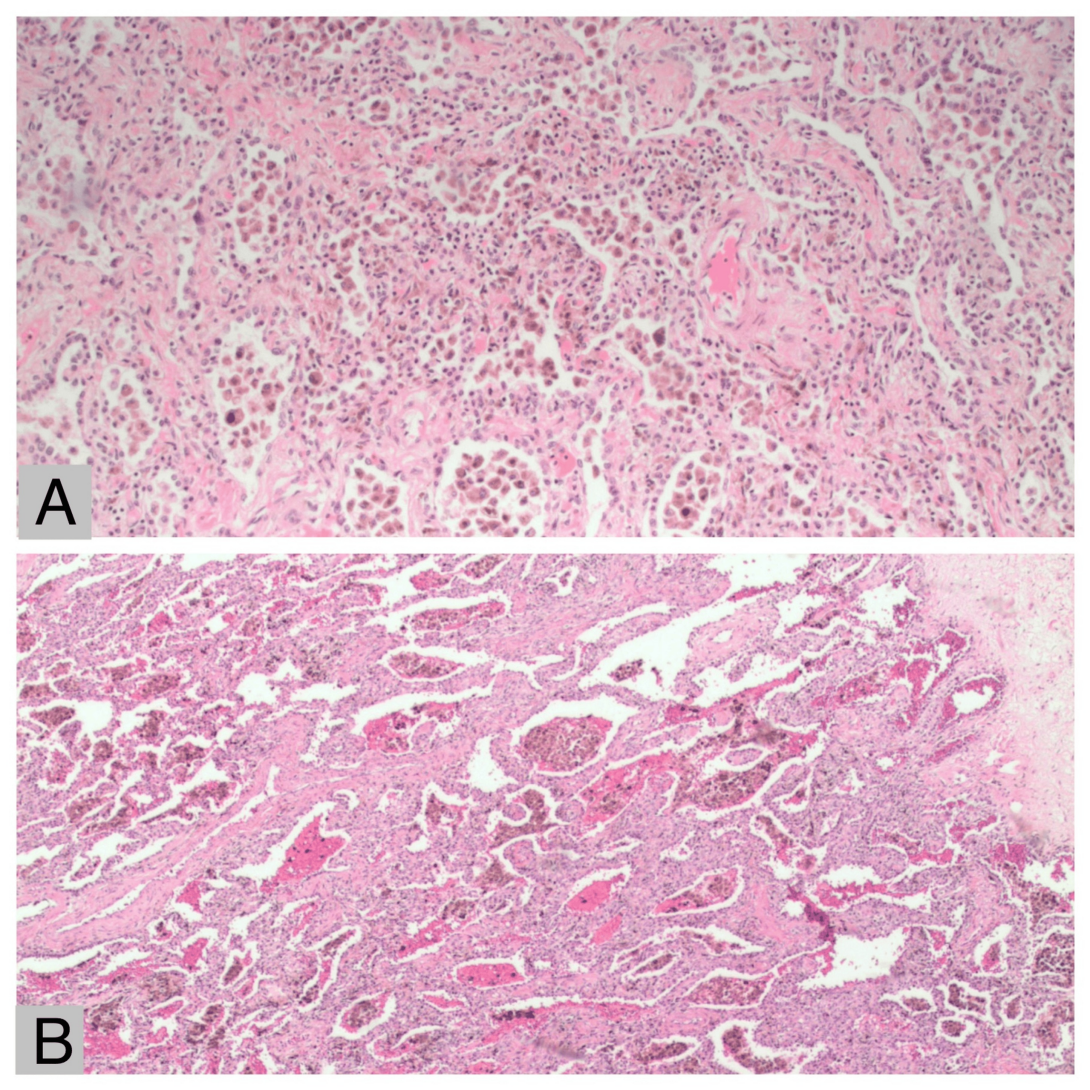
Histopathological examination of the lung specimen This image shows H&E staining of the lung tissue under different magnifications as follows: (A) The lung resection showed widespread bleeding and macrophages filled with hemosiderin as shown under 4X magnification. (B) The lung resection showed widespread bleeding and macrophages filled with hemosiderin as shown under 10 X magnification.

Upon readmission, the patient exhibited severe hypoxemia and elevated inflammatory markers. Imaging studies revealed bilateral patchy consolidations (Figure 1). Suspecting COVID-19, treatment was initiated with steroids, broad-spectrum antibiotics, and remdesivir. Despite this, the patient’s clinical condition deteriorated, necessitating intubation. Within one week of intubation, worsening respiratory failure with Arterial Blood Gas values, as shown in Table 1, prompted the initiation of veno-venous extracorporeal membrane oxygenation (VV ECMO) support via bilateral femoral cannulations. Due to persistent oxygenation challenges over the following weeks, the circuit was transitioned to a right internal jugular ProtekDuo system (LivaNova, London, UK). Despite aggressive interventions, the patient remained dependent on ECMO and experienced progressive clinical decline. An extensive workup, including diagnostic bronchoscopy, was performed to rule out conditions such as aspergillosis, blastomycosis, histoplasmosis, malignancy, and sarcoidosis. While awaiting results, the patient developed multiple complications, including Enterococcus sepsis, *Escherichia coli *(E. coli) bacteremia, heparin-induced thrombocytopenia (HIT), anemia, ileus, and wide complex tachycardia. Bronchoscopic findings revealed histological features (Figure 2) consistent with non-specific interstitial pneumonia (NSIP), diffuse alveolar hemorrhage, and pulmonary hypertension. The lungs showed widened alveolar septae due to chronic inflammation and mild fibrosis, reactive type 2 pneumocytes lining the septae, and intra-alveolar macrophages containing hemosiderin and hematoidin pigments. Scattered foreign body macrophages with talc-like material, organizing pneumonia, fibrin balls within alveoli, peribronchiolar scars, dilated/thickened vessels, and a 9 mm hematoma in the left upper lobe were also observed.

**Table 1:** Arterial Blood Gas Values The arterial blood gas (ABG) results show worsening respiratory status while intubated, prompting the need for veno-venous extracorporeal membrane oxygenation (VV ECMO). PCO2: partial pressure of carbon dioxide; PO2: partial pressure of oxygen; HCO3: bicarbonate; Est O2 SAT: estimated oxygen saturation; FiO2: fraction of inspired oxygen

pH	7.46
PCO2	4.06
PO2	9.01
HCO 3	23.1
Est O2 SAT	90.6%
FiO2	28%

Due to the severity of his respiratory failure, complications, and no improvement in his condition, the patient was then transferred to a different hospital for evaluation for lung transplantation. Before undergoing a lung transplant, he experienced significant nasopharyngeal bleeding. This issue was resolved after Interventional Radiology and Ear Nose Throat (ENT) specialists performed a therapeutic bronchoscopy. The patient then underwent a successful bilateral lung transplant and was subsequently transferred to the Cardiovascular Intensive Care Unit (CVICU), after which the diagnostic bronchoscopy findings were confirmed as well. 

**Figure 3:**
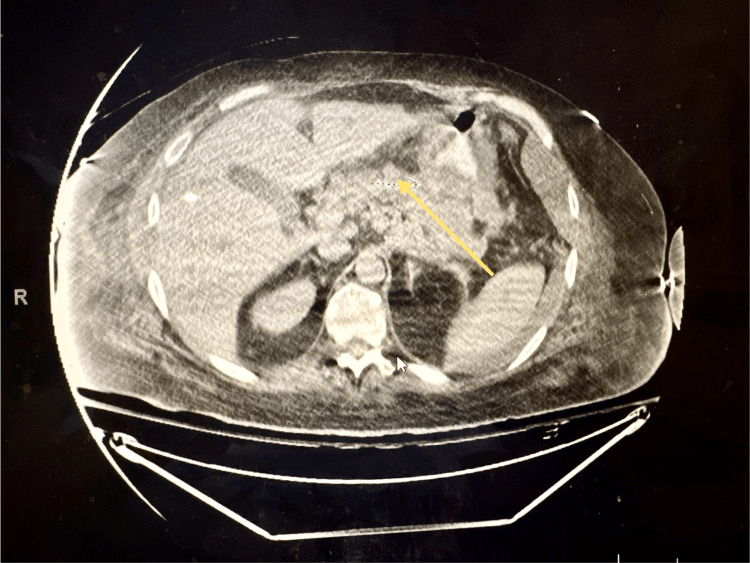
CT scan of Abdomen This imaging shows areas very small low attenuation within the pancreatic parenchyma, indicating necrosis, accompanied by high attenuation areas representing blood collections within the pancreas and surrounding tissue, signifying hemorrhage

Following the procedure, he received bifemoral VV ECMO and nitric oxide support. Vasopressors were initiated due to hypotension. After about a day, his oxygenation improved, leading to the removal of VV ECMO within the next 24 hours. Approximately two weeks post-transplant, the patient developed sepsis with acute pancreatitis, necessitating urgent laparotomy, gastrojejunal tube placement, and drain insertion. At four weeks, respiratory complications arose, requiring bronchial stent placement and interventions for lung and anastomotic wound dehiscence. By six weeks, acute hemorrhagic pancreatitis with active extravasation was identified (Figure 3), but despite angiography and embolization, the patient’s condition deteriorated. At seven weeks, care was withdrawn, and the patient passed away.

## Discussion

Diffuse alveolar hemorrhage (DAH) is a rare, potentially life-threatening complication, characterized by bleeding in alveolar spaces, leading to respiratory failure. It is associated with a variety of infections including COVID-19 and noninfectious conditions like autoimmune diseases, connective tissue diseases, vasculitis, drug toxicities, and radiation-induced damage. Patients with diffuse alveolar hemorrhage typically manifest with several distinguishing features, including cough, dyspnea, hemoptysis, anemia, diffuse alveolar infiltrates on imaging, and hypoxemic respiratory failure [4].

The pathogenesis of DAH following COVID-19 infection involves endothelial damage and an inflammatory response triggered by the virus. SARS-CoV-2 binds to the angiotensin-converting enzyme 2 (ACE2) receptor, which is extensively expressed in pulmonary vessels and increases vascular permeability and bleeding into the alveolar spaces [5]. Moreover, COVID-19 is also associated with a hyperinflammatory state known as cytokine storm which is characterized by elevated levels of systemic Inflammatory cytokines and biomarkers, including granulocyte colony-stimulating factor, interleukins (e.g., IL-2, IL-6, and IL-7), macrophage inflammatory protein 1-a, tumor necrosis factor (TNF)-a, C-reactive protein, ferritin, and D-dimer [6]. These increased inflammatory cytokines disrupt the alveolar-capillary basement membrane and result in hemorrhage.

The preferred method for diagnosing DAH is flexible bronchoscopy with bronchoalveolar lavage, which should be carried out immediately for early diagnosis. DAH presents a typical histopathological pattern on biopsy i.e. hemosiderin-laden macrophages, fibrin, or red blood cells (RBCs) in the alveolar space [7]. Once DAH is diagnosed, the underlying cause is investigated by a thorough history, regarding drug use, toxic or viral exposures, extrapulmonary symptoms, physical examination, relevant laboratory testing, and imaging.

Management of diffuse alveolar hemorrhage is based upon identifying and treating the underlying cause such as addressing infections, stopping causative medications and eliminating the associated exposures, and reversing the excessive anticoagulation. Inflammation associated with DAH should be managed with the administration of systemic glucocorticoids, and immunosuppressive medication (such as rituximab or cyclophosphamide), with or without plasmapheresis [8]. For, hypoxemic respiratory failure, supportive care is provided by supplemental oxygen, non-invasive ventilation, and mechanical ventilation depending upon severity. Extracorporeal membrane oxygenation (ECMO) can also be used in patients with refractory hypoxemic respiratory failure due to DAH [9]. Despite all these interventions, DAH has a poor prognosis with mortality rates ranging from 20% to 50% [10].

We conducted a comprehensive literature review, summarized in (Table 2), focused on case reports involving patients with diffuse alveolar hemorrhage following covid 19 infection. A total of 12 cases, including seven males and five females, with ages ranging from 26 to 79 years, were identified. Five of them had associated autoimmune diseases like microscopic polyangiitis and granulomatosis with polyangiitis. Mortality was high among these patients despite aggressive interventions, highlighting the importance of early diagnosis and prompt management of diffuse alveolar hemorrhage in COVID-19 patients.

**Table 2:** Summary of reviews of the case reports of COVID-19 patients with diffuse alveolar hemorrhage and outcomes

Authors	Age	Gender	Presentation	Associated Autoimmune Disease	Covid Vaccination status	Outcome
Mohammadi et al, 2021 [11]	59	Male	Cough, Low-Grade fever	Unknown	Unknown	Discharged
Wali et al, 2021 [8]	26	Female	Shortness of breath, Dyspnea on exertion	Microscopic Polyangiitis	Unknown	Discharged
Löffler et al, 2019 [4]	79	Male	Generalized malaise, Non-productive cough	Negative	Unknown	Death
Löffler et al, 2019 [4]	70	Male	Malaise, Intermittent fever	Negative	Unknown	Discharged
Takahashi et al, 2023 [12]	30	Female	Fever, Dyspnea	Negative	Unvaccinated	Death
Peys et al, 2020 [13]	58	Male	Haemoptysis	Negative	Unknown	Discharged
Patel et al, 2021 [14]	77	Female	Fever, Dyspnea, Hemoptysis	Microscopic Polyangiitis	Unknown	Death
Seng et al, 2022 [15]	33	Male	Hemoptysis, Dyspnea	Unknown	Unknown	Discharged
Siddiqui and Tchakarov, 2024 [16]	78	Female	Shortness of breath and Hemoptysis	Negative	Unknown	Death
Lind et al, 2021 [17]	40	Male	Diffuse Myalgias, Fever, and congestion.	Granulomatosis with polyangiitis	Unknown	Discharged
Hussein et at, 2020 [18]	37	Female	Chest pain, cough, shortness of breath, hemoptysis	Granulomatosis with polyangiitis	Unknown	Death
Assar et al, 2021 [19]	67	Male	Fever, malaise, dry cough	Microscopic polyangiitis	Unknown	Discharged

## Conclusions

This case highlights the severe complications of COVID-19 in an unvaccinated individual, particularly the development of diffuse alveolar hemorrhage (DAH). Despite aggressive interventions, including intubation, ECMO, and a bilateral lung transplant, the patient’s condition continued to worsen, ultimately leading to death. This tragic outcome underscores the vital importance of vaccination in preventing severe COVID-19 and its potentially life-threatening complications. Furthermore, the case points to the urgent need for ongoing research to identify more effective treatment strategies for critically ill patients. While newer therapies such as Tocilizumab, Regeneron, and Sarilumab have shown promise, they were discovered and became available only after this patient’s death. This highlights the need for continuous exploration of novel treatments and better data to improve the management of patients with severe COVID-19.
